# Estimating koala density from incidental koala sightings in South‐East Queensland, Australia (1997–2013), using a self‐exciting spatio‐temporal point process model

**DOI:** 10.1002/ece3.8082

**Published:** 2021-09-17

**Authors:** Ravi Bandara Dissanayake, Emanuele Giorgi, Mark Stevenson, Rachel Allavena, Joerg Henning

**Affiliations:** ^1^ School of Veterinary Science The University of Queensland Gatton Qld Australia; ^2^ Lancaster Medical School Lancaster University Lancaster UK; ^3^ Faculty of Veterinary and Agricultural Sciences University of Melbourne Parkville Vic. Australia

**Keywords:** citizen science, koala, modeling, population, Queensland

## Abstract

The koala, *Phascolarctos cinereus*, is an iconic Australian wildlife species facing a rapid decline in South‐East Queensland (SEQLD). For conservation planning, the ability to estimate the size of koala populations is crucial. Systematic surveys are the most common approach to estimate koala populations but because of their cost they are often restricted to small geographic areas and are conducted infrequently. Public interest and participation in the collection of koala sighting data is increasing in popularity, but such data are generally not used for population estimation. We modeled monthly sightings of koalas reported by members of the public from 1997 to 2013 in SEQLD by developing a self‐exciting spatio‐temporal point process model. This allowed us to account for characteristics that are associated with koala presence (which vary over both space and time) while accounting for detection bias in the koala sighting process and addressing spatial clustering of observations. The density of koalas varied spatially due to the heterogeneous nature of koala habitat in SEQLD, with a mean density of 0.0019 koalas per km^2^ over the study period. The percentage of land areas with very low densities (0–0.0005 koalas per km^2^) remained similar throughout the study period representing, on average, 66% of the total study area. The approach described in this paper provides a useful starting point to allow greater use to be made of incidental koala sighting data. We propose that the model presented here could be used to combine systematic koala survey data (which is spatially restricted, but more precise) with koala sighting data (which is incidental and often biased by nature, but often collected over large geographical areas). Our approach could also be adopted for modeling the density of other wildlife species where data is collected in the same manner.

## Graphical abstract

A self‐exciting spatio‐temporal point process model was developed to estimate koala density from observed koala sightings data while accounting for spatio‐temporal detection bias and clustering of observations. An example of koala densities between 1997 and 2006, estimated from koala sightings data is shown below.

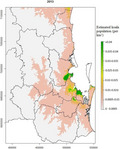

## 1 INTRODUCTION

Since the time of first European settlement in Australia, koalas have faced threats from humans. Threats have included hunting (until 1927 in Queensland) (Melzer et al., 2000) and fragmentation of habitat due to urbanization. An excess of koala mortalities occurs from traumatic injuries from vehicular collisions and dog attacks or diseases such as chlamydia (Adams‐Hosking et al., 2011; Lunney et al., 2002; Preece, 2007). Over the past three decades, the koala population in Australia experienced a decline of 24% (Adams‐Hosking et al., 2016). To inform koala conservation efforts, it is essential to know the geographic distribution of koala habitat and koala population densities (Adams‐Hosking et al., 2016; Ellis et al., 2013). While identification of suitable koala habitat areas is relatively straightforward, the estimation of koala densities in habitat suitable areas is a difficult task (MacKenzie, 2006). Usually, systematic sampling methods are used to count koalas and estimate koala population densities. These methods typically follow a defined approach to collect data from areas that are thought to be representative of the entire geographic area of interest. These include transect and distance sampling methods (Crowther et al., 2020; Dique et al., 2004; Wilmott et al., 2019) which require skilled observers to identify and count koalas (Thomas et al., 2010). For this reason, they are labor‐intensive and expensive (Kjeldsen et al., 2015) and therefore only able to be carried out over small areas.

Research has described the relationship between koala tree preference and the presence of scat (Ellis et al., 2013), and koala scat prevalence has been shown to correlate well with koala population densities (Ellis et al., 1998; Lunney et al., 2009; McAlpine et al., 2006; Phillips & Callaghan, 2011; Rhodes et al., 2008). To monitor the impact of koala conservation efforts, it is important to generate long‐term datasets of koala population estimates over large geographical areas rather than estimate population counts in smaller areas at infrequent intervals (Ellis et al., 1998; Lunney et al., 2014, 2016). Appropriate ecological and statistical modeling techniques can help inform decision‐making in wildlife conservation if empirical data are limited (Schmolke et al., 2010) and account for biases associated with the data collection method used. A recent study in South‐East Queensland (SEQLD) attempted to estimate the geographic distribution of koala populations across a wide geographical area by using a statistical modeling approach to account for multiple survey methods and multiple observers and errors in the data collection process using transect survey data collected between 1996 and 2015 (Rhodes et al., 2015).

With the advancement of communication technologies and the widespread availability of dedicated mobile applications, public participation in collecting wildlife data is increasing in popularity. For instance, members of the public were invited to collect koala sighting data as part of a program titled the “Great Koala Count” in the Australian states of New South Wales and South Australia in 2012 (Sequeira et al., 2014). The Great Koala Count has generated a large amount of incidental koala sighting data using specific guidelines for data collection in preidentified geographical areas in these states. Attempts have been made to estimate wildlife populations using incidental sighting data alone and/or in combination with survey data (Dorazio, 2014; Sequeira et al., 2014).

In SEQLD, incidental koala sighting data have been collected since 1997, although no formal field protocols are provided to members of the public to ensure that recorded observations are valid (Dissanayake et al., 2019). While these data have been used to describe trends in sighting frequency and document spatial biases associated with sighting data (Dissanayake et al., 2019), they have not, to the best of our knowledge, been used to estimate koala population densities. It had been shown that koala sightings are spatially biased toward roads (where human activity is more frequent) and sightings tend to be more common during koala breeding seasons (Dissanayake et al., 2019, 2021). In this study, we use SEQLD incidental koala sighting data collected over a period of 17 years to develop a modeling approach to estimate koala density, accounting for spatio‐temporal detection biases and biases arising from geographic clustering of observations.

## 2 MATERIALS AND METHODS

### 2.1 Study area

**FIGURE 1:**
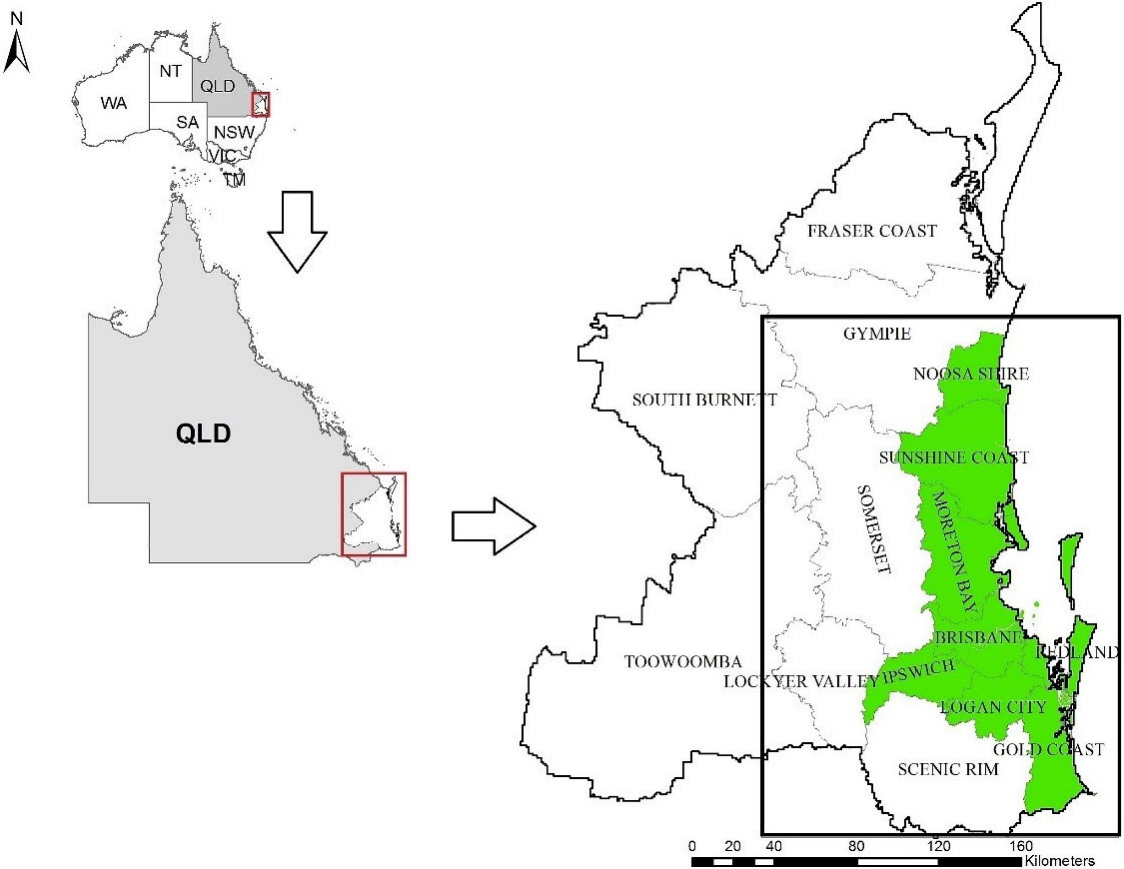
Map showing the location of the study area in South‐East Queensland. The superimposed rectangular land area defines the boundaries of the area used for data analysis (water areas were excluded from the analysis). The areas marked in green are the main koala conservation areas

The geographical area of interest for this study comprised 15 local government areas (LGAs) in SEQLD (Figure 1). South‐East Queensland has a higher population of koalas compared with other areas of Queensland (Dique et al., 2004) but also has a relatively high level of urban development. The point locations of koala sighting events were plotted on a map of SEQLD, indicating relatively few sightings in the north and to the far west of the candidate study area. To develop an observation window that was tractable for modeling, we constructed a convex hull around all koala sighting locations and then dilated the convex hull by 3 km to accommodate the home range of koalas sighted on the border of the observation window (de Oliveira et al., 2014). Refinement of the study area in this way reduced the number of constituent LGAs from 15 to 11 and included parts of three remaining LGAs. The selected LGAs were in the eastern and central parts of SEQLD. The northern LGA of the Fraser Coast was excluded due to an absence of koala sighting records. This reduced the study area from 57,800 square kilometers to 30,500 square kilometers.

### 2.2 Koala sighting data

Our definition for a koala sighting was a live koala observed and reported to the Queensland Department of Environment and Science (DES) by a member of the public, which we call an “incidental sighting” in the remainder of this paper. Incidental koala sightings reported in SEQLD between 1997 and 2013 (*n* = 14, 250 sightings) were retrieved from KoalaBASE, a database of clinical admission and sighting data of koalas, developed by the University of Queensland's School of Veterinary Science (www.koalabase.com.au) and managed by DES (https://environment.des.qld.gov.au/). The dataset for analysis included the date of each sighting event as well as the longitude and latitude of the location where the koala was actually observed.

We assumed that if two koalas were reported in the same area in the same month, these could possibly be repeated sightings of the same animal. The term “area” in this context represents the estimated home range for a koala, which was assumed to be circular with an average (*A*) size of 0.35 km^2^ (de Oliveira et al., 2014). We developed a selection process in which we permitted a sighted koala to be seen in the same area a maximum of 12 times per year in an attempt to remove duplicate records arising from observation of the same koala at different times (i.e., this resulted in the selection of one sighting location per koala per month). To do this, we assumed the minimum distance between two koalas was given by $d=2\times \sqrt{A÷\pi }$, or 666 m. Observations selected per month had a minimum distance of 666 m between closest neighbors, but observations across months did not necessarily have this minimum distance to acknowledge that home ranges can overlap over time (Ellis et al., 2009).

**FIGURE 2:**
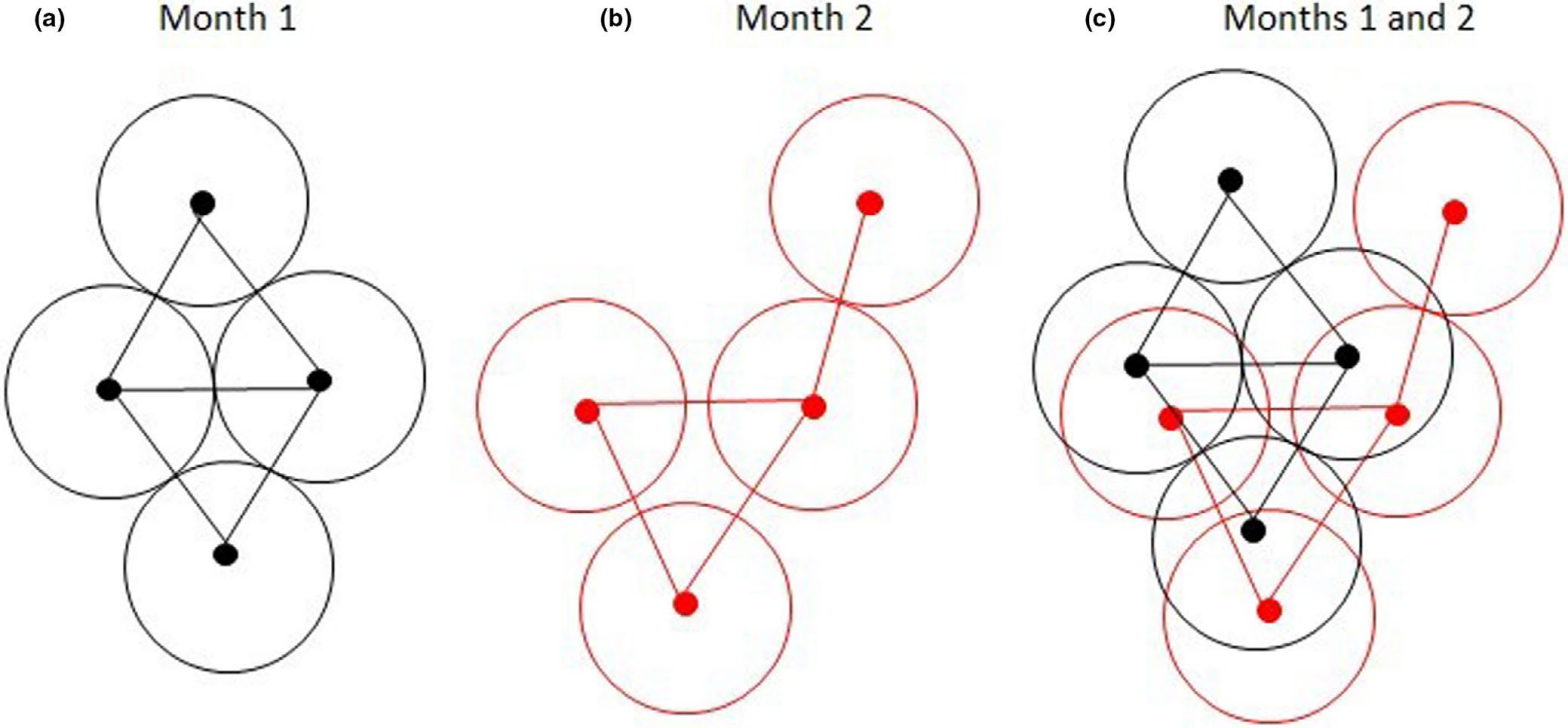
Diagram showing the method used to select incidental koala sightings to estimate koala population density. Circles represent an average home range area for a koala. Black and red dots represent locations of koala sightings for month 1 and month 2, respectively. The black and red lines show the closest distance between two koala sighting locations. (a) shows a selection of koala sighting locations for month 1, while (b) shows a selection of koala sightings for month 2. (c) shows the two monthly datasets combined. Using this approach, monthly sightings for each year over the 17‐year study period were compiled and used to estimate koala sighting density

Subsequently, ensuring this minimum distance between two koala locations per month, the sightings for each month over the 17 years were compiled, resulting in 12 months × 17 years = 204 sighting months combined into a single dataset (Figure 2).

### 2.3 Spatio‐temporal modeling of koala sighting density

The density of koala sightings (providing a proxy for koala population density) was estimated as a realization of a spatio‐temporal point process model (STPP) model (Baddeley et al., 2016) using koala sighting presence‐only data. The STPP is a useful statistical tool that allows one to model the spatial and temporal variation of sightings within a region and time period of interest. Within this modeling framework, two main methods can be distinguished: (a) mechanistic models, where subject matter knowledge is used (i.e. our current knowledge about koala habitat and koala behavior to select relevant spatial covariates, to decide an average home range of koalas, and to describe the seasonality or “clustering” of sightings in the mating versus non‐mating period) to inform the probability that a sighting will occur at a particular location and a particular time point, and (b) empirical models, where the objective is to use the observed data to inform estimates of koala population density. The approach described in this paper uses a combination of the two methods.

More specifically, we assumed that the koala density at a particular location and a particular time was dependent on the three groups of variables: (a) variables that relate to spatio‐temporal detection bias; (b) spatio‐temporally referenced variables that are known to be associated with koala density; and (c) variables that reflect well‐established knowledge on the home range of koalas. The main mechanistic component of the model is provided by the third group of variables, while the first two are modeled as a log‐linear regression of koala density. A partial likelihood approach was used to fit the model (Diggle et al., 2010).

Two main factors were considered in the spatio‐temporal modeling of koala sighting density: spatio‐temporal detection bias $b\left(x,t\right)$ and the true koala density given by $\lambda_{o}\times q\left(x,t\right)\times r\left(x,t|H_{t}\right)$, where $q\left(x,t\right)$ represents the effect of observed spatio‐temporal variables, $r(x,t|H_{t})$ is the spatial interaction between koalas, and $\lambda_{o}$ is an intercept term (and where t denotes year and x spatial location).

Let $H_{t}$denote the past history of the process (i.e., all koala sightings) up to year t. By observing $H_{t}$, we condition on all the koala sightings recorded up to year tand define the intensity λof the process at a location xin year tas follows:

(1) $$\lambda (x,t|H_{t})=\lambda_{o}\times q\left(x,t\right)\times r\left(x,t|H_{t}\right)\times b\left(x,t\right)$$

Here, we refer to $\lambda (x,t|H_{t})$ as the koala sighting density or, in other words, the expected number of sightings per square kilometer per year. We then denote the product $\lambda_{o}\times q\left(x,t\right)\times \;r\left(x,t|H_{t}\right)$, which excludes the bias $b\left(x,t\right)$as the (true) koala density. Intuitively, if the bias $b\left(x,t\right)=1$ for all locations and years, then $\lambda (x,t|H_{t})$ and the true koala density $\lambda_{o}\times q\left(x,t\right)\times \;r\left(x,t|H_{t}\right)$ would coincide; if, instead, $b\left(x,t\right)$< 1 or $b\left(x,t\right)$> 1, these would imply underestimation and overestimation of the true koala density, respectively. The term q(x,t)that incorporates the covariates that influence koala density only and the spatio‐temporal detection bias b(x,t) were modeled as a log‐linear regression on covariates e(x,t) and $d\left(x,t\right)$, respectively.

(2) $$\log\left\{q\left(x,t\right)\right\}=\;\gamma^{⊺}e\left(x,t\right);$$

(3) $$\log\left\{b\left(x,t\right)\right\}=\beta^{⊺}\;d\left(x,t\right)$$

In this analysis, we used the covariate *distance to primary roads* as d(x,t) and used all the other covariates, which we describe more in detail in the next section as e(x,t).

The third factor corresponds to clustering of koalas that cannot be explained by $d\left(x,\;t\right)\;\mathrm{and}\;e\left(x,t\right)$ was modeled as follows:

(4) $$r\left(x,t|H_{t}\right)=\prod_{j:t_{j=t‐1}}\{1+\left[\theta \left(t\right)‐1\right]f(||x‐x_{j}\left||)\right\},$$

(5) $$f\left(||x‐x_{\mathrm{ij}}\left|\right|\right)=\left\{\;\begin{array}{c} \exp\{‐||\;\;x‐\;x_{j}||/\phi \;\;\;\;\;if\;||x‐x_{j}||\;\;<u\left(x\right)\\ 0\;\;\;\;\;\;\;\;\;\;\;\;\;\;\;\;\;\;\;\;\;\;\;\;\;\;\;\;\;\;\;\;\;\;\;\;\;\;\;\;\;\;\;\;\;\;\;\;if\;||x‐x_{j\;}\left|\right|,\;\;\geq u\left(x\right) \end{array}\right.$$

In Equation 4, the parameter θ(t) regulates the strength and direction of the spatial interaction between koala sightings: 0<θ(t)<1 represents an inhibitory point process; θ(t)=1 is the case of no interaction, and θ(t)>1 represents the situation where koala sightings are spatially aggregated. In Equation 5, u(x) is the square root of the koala home range (in kilometers squared) at location x and ϕ is a scale parameter that regulates how quickly the spatial interaction between koalas decays as a function of distance $||x‐x_{j\;}\left|\right|$. We let θ(t) vary between the mating and non‐mating seasons as we expected a stronger spatial interaction of koalas and thereby potential aggregations of sightings during the former period. Hence, $\theta_{2}$ was used to denote the strength of the spatial interaction during the mating season (August to September, de Oliveira et al., 2014) and $\theta_{1}$ the spatial interaction parameter for other times of the year.

Let i denote the ith koala sighting. We fit the model using the partial likelihood ($L_{p}$) function based the koala sightings reported during the observation period of 17 years:

(6) $$L_{p}=\sum_{i=1}^{N}\log\frac{q\left(x_{i},t_{i}\right)b\left(x_{i},t_{i}\right)r\left(x_{i},t_{i}|H_{t_{i}}\right)}{\int_{A}^{\cdot }b\left(x,t_{i}\right)q\left(x,t_{i}\right)r\left(x,t|H_{t_{i}}\right)dx}$$

In Equation 6, N is the total number of observed koalas throughout the study period. Note that the partial likelihood does not allow us to estimate the intercept $\lambda_{o}$ which is required to predict the density of koalas at any given time and location. Hence, after estimating all the model parameters using partial likelihood in Equation 6, we also estimate $\lambda_{o}$ as follows:

(7) $${\hat{\lambda }}_{0}=N{\left(\int_{A}^{\cdot }b\left(x,t_{i}\right)q\left(x,t_{i}\right)r\left(x,t|H_{t_{i}}\right)dx\right)}^{‐1}$$

Note that this estimator for the intercept is obtained by equating the total number of observed koalas N to the total number of expected koalas based on the model in Equation 7. To predict the density of koalas at a location x and time t, we use the expression ${\hat{\lambda }}_{0}q\left(x,t\right)r\left(x,t|H_{t}\right)$ from which the bias term $b\left(x,t\right)$ was excluded.

The likelihood function given by the above equation was maximized using a numerical optimization procedure implemented in the nlimnb function in R (version 3.5.1). Since the model described above was not available in any of the existing R packages, the algorithm used in this analysis was developed from first principles in R.

We present the model results as a graph of the predicted monthly koala population over the 17‐year study period and as maps of estimated koala population density (koalas per km^2^) for each year in SEQLD, 1997–2013. We also present a map of the estimated spatial detection bias. Although the aim of this study was to estimate spatio‐temporal koala density from sighting data, we also provide predicted coefficients for the covariates with 95% confidence intervals.

### 2.4 Covariates

The following data were used as explanatory variables in the model: distance to primary roads (meters), land lot density (number of lots per square kilometer), mean temperature of the hottest month per year (degrees Celsius), mean temperature of the coldest month per year (degrees Celsius), precipitation of the driest month per year (millimeters), precipitation of the wettest month per year (millimeters), mean elevation above sea level (meters), and foliage projective cover (proportion).

The variable *distance to primary roads* was considered to only influence spatio‐temporal bias associated with koala sightings (Dissanayake et al., 2019) since it provides a measure of observer access to koala habitat. The other variables were assumed to influence observed koala sightings as they represent the documented impact of climate (temperature, precipitation), environmental factors (foliage protective cover, elevation), and indicators for the presence of humans (land lot density) on koala populations.

All of the climatic variables were obtained from online spatial databases as raster maps recorded at 1 square kilometer resolution. The climate and elevation data were downloaded from WorldClim online database (http://www.worldclim.org) for the period 1950–2000 (Hijmans et al., 2005). Foliage protective cover represents the percentage of ground area occupied by the vertical projection of foliage for 2010 and was obtained from the Queensland Government spatial catalogue (https://qldspatial.information.qld.gov.au). Primary roads and land lot density were obtained as shapefiles from the same spatial database. Distance to primary roads was calculated from its shapefile. Both, distance to primary roads and land lot density were converted to raster maps.

We standardized each covariate by subtracting the mean and dividing it by the standard deviation.

The standardized covariates are presented as raster maps in Figure S1.

## 3 RESULTS

**FIGURE 3:**
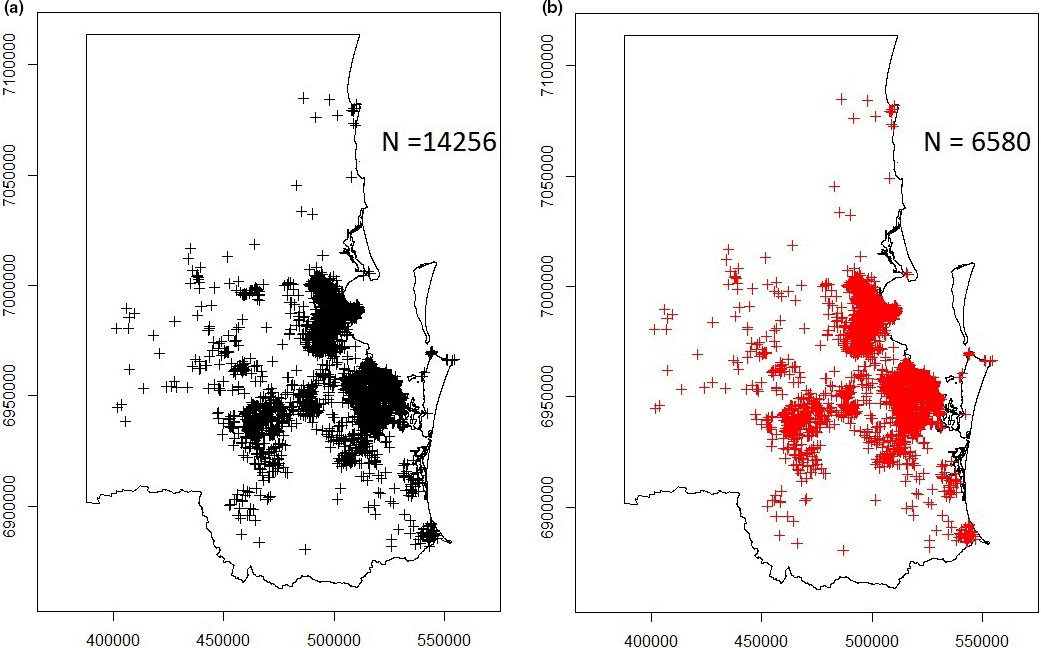
Maps of the study area in South‐East Queensland, Australia, showing: (a) the point location of all incidental koala sightings recorded in KoalaBASE between January 1997 and December 2013 (n = 14,256) and (b) the point location of incidental koala sightings used for analysis (n = 6,580)

The described approach of selecting monthly sightings over the 17‐year observation period resulted in the reduction in the original dataset comprised of 14,256 sightings to a dataset comprised of 6,580 sightings. The total number of koala sightings and the subset of sightings used for analysis are shown in Figure 3a,b, respectively.

**FIGURE 4:**
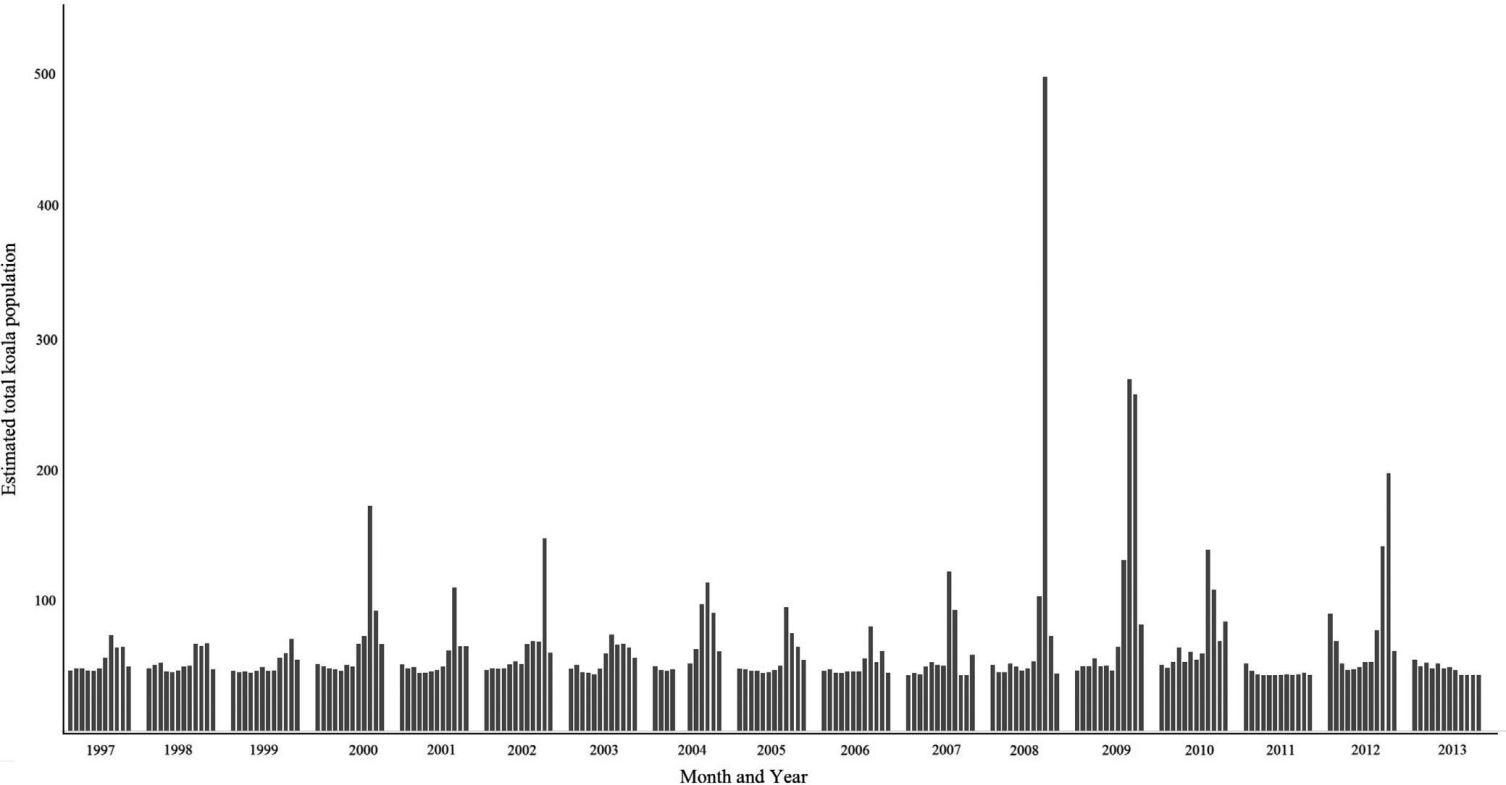
Bar graph showing the estimated total number of koalas in the South‐East Queensland study area estimated from the spatio‐temporal point process model as a function of calendar month and year. The spatio‐temporal point process model has been informed by monthly observed koala sightings (n = 6,580) recorded in South‐East Queensland, 1997–2013

A bar graph showing the estimated total monthly koala population by calendar time estimated from the spatio‐temporal point process is shown in Figure 4. The koala population was low for the period 1997–1999 without prominent peaks, then fluctuated with peaks from 2000 to 2007 (with biennial larger peaks), before reaching large seasonal peaks in 2008 and 2009, declining again to peaks similar to pre‐2008 period, followed by another large peak in 2012.

Parameter estimates from the spatio‐temporal point process model used to estimate koala population densities in SEQLD between 1997 and 2013 are shown in Table S1. The coefficients of land lot density and mean temperature of the coldest month were positive, while the coefficients of the other covariates included in the model were negative. The estimate of the intercept was ${\hat{\lambda }}_{0}=0.006$.

**FIGURE 5:**
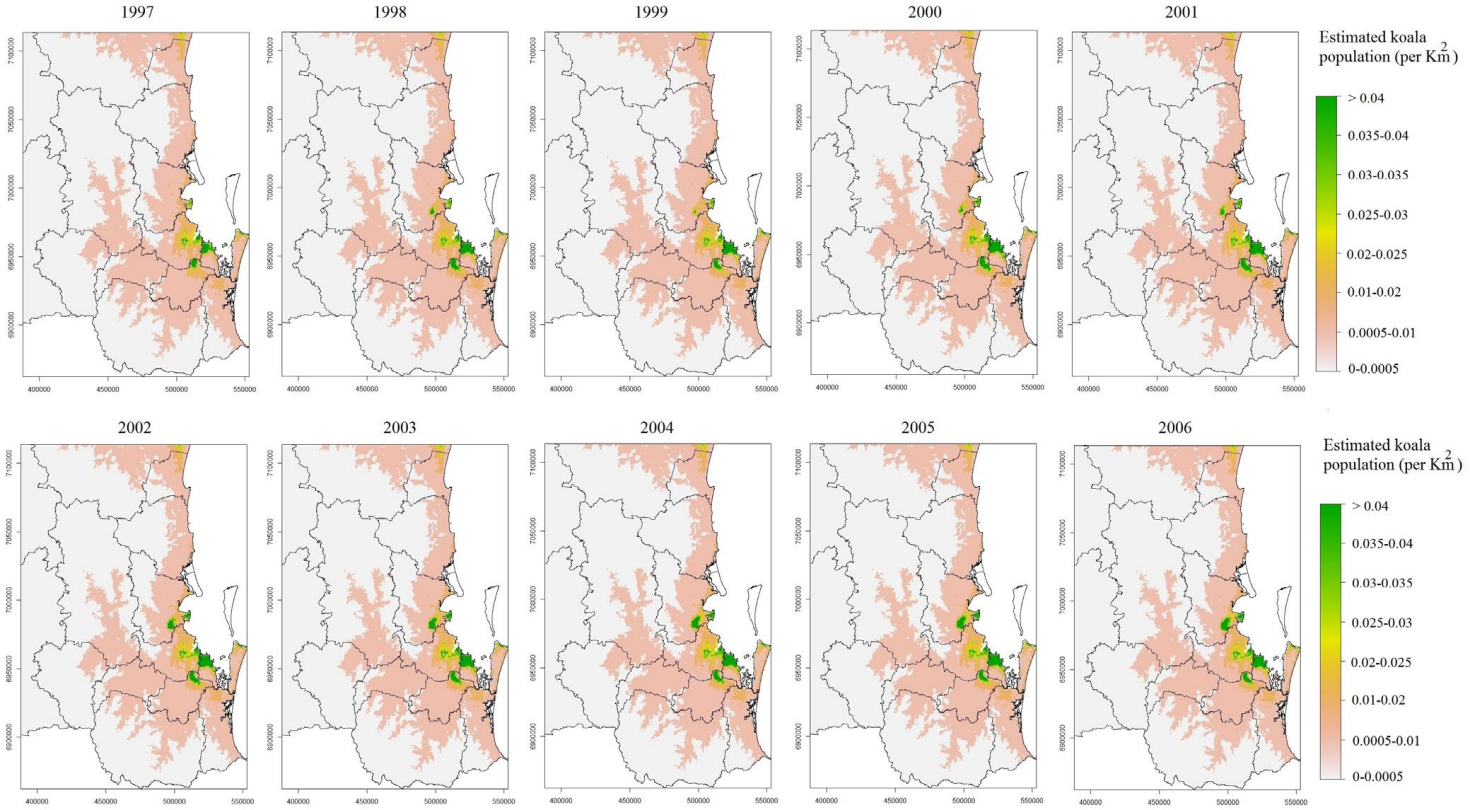
Raster map showing estimated koala population densities (koalas per km^2^) across South‐East Queensland between 1997 and 2006. Estimates were derived from a spatio‐temporal point process model using koala sighting data (n = 6,580)

An example raster map showing the estimated koala population density (koalas per km^2^) across SEQLD between 1997 and 2006 is shown in Figure 5. The estimated koala population density in SEQLD across the whole study period (1997‐2013) is shown in Figure S2 with a scale ranging from 0 to 0.04 or more koalas per km^2^. The predicted density of koalas in the study region varied throughout the study period, with a mean density estimate of 0.0019 koalas per km^2^ over the 1997–2013 period (with a maximum of 0.69 kolas per km^2^), although estimated density in the same areas remained more or less the same throughout the study period.

Based on the model presented in Equation 6, θ was >0 and φ was small suggesting that koalas aggregate over relatively large areas. The predicted spatial distribution of koalas is consistent with the observed sighting data, with no koalas or very low koala densities in the western part of the study area and koala densities increasing toward the eastern coast of SEQLD, with prominent pockets of relatively high koala densities in areas with (known) good koala habitat.

The percentage of the study area in SEQLD with categorized koala sighting densities (koalas per km^2^) for each year of the 1997–2013 study period are shown in Table 1. The percentage of land areas with very low sighting densities (0–0.0005 koalas per km^2^) remained similar throughout the study period representing a mean (*SD*) of 66% (0.06) of the total study area (Table 1). However, land areas with more koalas per km^2^ showed larger variations over the years, with koala mean (*SD*) densities of 0.0005–0.01, 0.01–0.02, 0.02–0.025, 0.025–0.03, 0.030–0.035, 0.035–0.040, and >0.4 koalas per km^2^ representing 30% (0.0), 2.7% (0.04), 0.55% (0.33), 0.22% (0.02), 0.18% (0.02), 0.12% (0.22), and 0.45 (0.09)% of the study area in SEQLD, respectively.

**TABLE 1:** Percentage of the South‐East Queensland study area, 1997–2013, belonging to each category of koala population density (koalas per km^2^)

Density^a^	Percent of land area																
	1997	1998	1999	2000	2001	2002	2003	2004	2005	2006	2007	2008	2009	2010	2011	2012	2013
0–0.0005	66	66	66	66	66	66	66	66	66	66	66	66	66	66	66	66	66
0.0005–0.01	30	30	30	30	30	30	30	30	30	30	30	30	30	30	30	30	30
0.010–0.020	2.7	2.8	2.8	2.7	2.7	2.8	2.7	2.7	2.7	2.7	2.7	2.7	2.7	2.7	2.7	2.7	2.7
0.020–0.025	0.50	0.53	0.51	0.55	0.54	0.56	0.61	0.62	0.61	0.58	0.58	0.53	0.56	0.56	0.55	0.55	0.54
0.025–0.030	0.20	0.23	0.23	0.22	0.22	0.20	0.19	0.21	0.23	0.26	0.24	0.25	0.21	0.21	0.22	0.23	0.22
0.030–0.035	0.16	0.16	0.15	0.15	0.17	0.19	0.20	0.20	0.20	0.18	0.19	0.19	0.19	0.18	0.19	0.18	0.20
0.035–0.040	0.07	0.10	0.10	0.13	0.15	0.11	0.11	0.11	0.11	0.11	0.15	0.13	0.16	0.14	0.13	0.13	0.12
>0.04	0.22	0.34	0.30	0.43	0.41	0.48	0.48	0.50	0.49	0.47	0.45	0.49	0.51	0.54	0.52	0.54	0.54

Note

Estimates were derived from a spatio‐temporal point process model using koala sighting data (n = 6,580).

^a^ Number of koalas per km^2^.

## 4 DISCUSSION

We present here the results of spatio‐temporal point process model where koala population density was estimated from citizen science koala sighting data by adjusting for spatio‐temporal detection bias.

The mean estimated density of koalas in the study region over the 1997–2013 period was 0.0019 koalas per km^2^ (with up to 0.69 koalas per km^2^). There was marked variation in koala population density largely due to the heterogeneous nature of koala habitat across the study area. Rhodes et al. (2015) reported koala densities ranging from 0.00001 to 0.11 per km^2^ in coastal regions of SEQLD, with an average of 0.0004 koalas per km^2^. Our study shows similar distribution patterns to the study by Rhodes et al. (2015), with high densities along the east coast area and two very high‐density spots in the central east coast area and low densities in the western part of the study area (Dissanayake et al., 2021). However, the model developed by Rhodes et al. (2015) used data collected through multiple systematic surveys, which were implemented in small areas and did not predict koala populations across large geographic areas due to uncertainties associated with extrapolation. In fact, extrapolating koala densities from statistical models for large geographical areas is questionable as koala habitat is not continuously distributed. Ideally, to avoid this problem, densities should be predicted to strata of different habitat types (Dique et al., 2004), but this was beyond the scope of the methodology presented in the Rhodes et al. (2015) paper.

Actual koala numbers are difficult to estimate. In 2010, the Department of Environmental Heritage and Protection (DEHP) predicted that the Queensland koala's population was between 157,000 and 177,000 individuals, while the Threatened Species Scientific Committee of Australia estimated Queensland's koala population to be approximately 167,000 individuals in 2010, representing a 43% decline from 1990 (Rhodes et al., 2015). Another study estimated Queensland's koala population to be about 79,300 in 2012 (Adams‐Hosking et al., 2016). Using expert elicitation methods, the koala population for the whole of Australia was approximated to be in the order of 329,000 individuals (range 144,000–605,000) (Adams‐Hosking et al., 2016). A decline of Queensland's koala population over the past 15 years, as estimated from expert opinion data (Adams‐Hosking et al., 2016), was not reflected in the results of our analyses. The fluctuation of the koala population over time might be a reflection of increased koala dispersal and movements during breeding seasons, but could also be related to increases in reporting in some years influenced by koala conservation initiatives or media reports on koala mortalities or, conversely, to events that reduced efforts made by members of the public to report koala sightings (e.g., flood events in 2011, Dissanayake et al., 2019).

We identified a strong clustering of koalas in locations in and around the Moreton Bay and Redlands areas which is similar to the high‐density areas identified by Rhodes et al. (2015) using systematic field survey data. Our model identified low densities of koalas in the western part of SEQLD whereas Rhodes et al. (2015) predicted higher densities there, although this was probably due to the uncertainty associated with the model estimates for this region.

Importantly, we were able to estimate koala population density over time and space while incorporating a range of covariates expected to be associated with observed sighting densities or spatio‐temporal detection bias. For example, distance to primary roads was considered to be an explanatory variable predominately influencing spatio‐temporal detection bias, while foliage protective cover was influencing an observer's ability to sight a koala and therefore impacting on observed sighting density. However, the contribution of explanatory variables to the two different components of the model cannot be quantified as these components were included as additive factors on a log‐scale. Considering that covariate coefficients with a negative sign decrease reported sightings, our model indicates that larger distances to primary roads, denser foliage, higher altitude and increased precipitation, results in decreased koala population density. In contrast, increased lot density and warmer temperatures in the colder months of the year were associated with increases in estimated koala population densities.

Uncertainties in estimated koala densities can be further reduced whether additional data are collected at the time of each sighting event. These data could then be used to estimate and remove the effect of the bias on population density estimates.

We have presented a map showing the geographic distribution of spatial detection bias (Figure S3). Due to the formulation of our model, detection bias is a function of the distance from a primary road, thus it does not change over time. Collecting additional data at the time of each sighting event (such as estimates of the experience of the individual making the observation, distance traveled, climate data) would allow search effort to be better quantified as additional terms in the model for b(x,t), Equation 3.

In our study, no observer‐related variables were collected at the time koalas were sighted. It has been shown that the probability of detection of a koala by an observer varies with their previous experience: An experienced observer can have a detection rate of around 70%, while an inexperienced observer might have a detection rate in the order of 30% (Corcoran et al., 2019). As a result, many koalas may go undetected simply because of the lack of observer experience. The situation is somewhat different in systematically conducted field surveys carried out by trained individuals (Rhodes et al., 2015). Thus, incidental sightings reported by members of the public represent a biased sample of the koala population at any given time, but the collection of data on the experience of observers at the time of the sighting could provide valuable information to correct for this bias.

The frequency of koala sightings varies between seasons of the year. Such seasonal variations might be due to more frequent dispersal of koalas during breeding periods but also due to better visibility of animals and weather conditions that are more favorable for people to go outdoors and spot koalas. Interestingly, the results of our model indicated that the clustering of koalas is not prominently different between the mating ($\theta_{1}$ = 2.006) and nonmating seasons of koalas ($\theta_{2}$ = 2.029). This might be explained by koalas being solitary animals, and although they travel over larger distances in the breeding season, their greater mobility might not necessarily be associated with clustering of animals.

We included an average home range of koalas in our model, as we did not have detailed koala home range information for different parts of our study area. We acknowledge that koala home ranges are not uniform, and even within the Redlands Local Government Area, koala home ranges of koalas vary between 0.05 and 0.55 km^2^ (de Oliveira et al., 2014). The precisions of koala densities could be improved if home ranges appropriate for each habitat type were included in the model.

It has been predicted that drier and warmer climatic conditions have an undesirable impact on koala habitat and thereby negatively impact on koala population densities (Adams‐Hosking et al., 2011). Unfortunately, our study was constrained by the nonavailability of some temporally varying explanatory variables (e.g., foliage projective cover and land lot density). Therefore, for consistency, we used a single value for each explanatory variable across all years, and, as a result, the temporal effect of explanatory variables such as the impact of temperature changes over time on koala densities could not be quantified.

In conclusion, we developed a statistical model that addressed the spatio‐temporal bias associated with observed koala sightings and provided long‐term koala density estimates for one of the largest koala populations of Australia over a 17‐year period. Such estimates of koala population size are required for koala population viability analyses, epidemiological models, translocation programs, and other koala management programs. In future, the modeling approach presented in this paper could be used to combine systematic (spatially restricted) survey data with koala sightings data, allowing koala population densities to be estimated over larger geographical areas. The systematic survey data could also provide the means by which to validate sightings data model outputs. Repeated systematic survey data in the same location could further inform the occupancy which can be used in an extended model. Our approach could also be adopted for modeling the density of other wildlife species where incidental sightings might be recorded.

## CONFLICT OF INTEREST

None declared.

## AUTHOR CONTRIBUTIONS

**Ravi Bandara Dissanayake:** Conceptualization (equal); formal analysis (equal); writing–original draft (equal). **Emanuele Giorgi:** Conceptualization (equal); formal analysis (equal); methodology (equal); writing–review and editing (equal). **Mark Stevenson:** Supervision (supporting); writing–review and editing (supporting). **Rachel Allavena:** Supervision (supporting); writing–review and editing. **Joerg Henning:** Conceptualization (lead); supervision (lead); writing–review and editing (supporting).

## Supporting information

Fig S1‐S3

Table S1

## Data Availability

The incidental koala sighting dataset analyzed in this study was obtained from KoalaBASE (www.koalabase.com.au).
