# Supplementary material for: Estimating koala density from incidental koala sightings in South‐East Queensland, Australia (1997–2013), using a self‐exciting spatio‐temporal point process model
Source: Ecol Evol. 2021 Sep 17;11(20):13805–14. doi: 10.1002/ece3.8082 (PMC8525080; doi:10.1002/ece3.8082)
Supplement: Supplementary file 1 — Fig S1‐S3 [file ECE3-11-13805-s001.docx]

**Estimating koala density from incidental koala sightings in South-East Queensland, Australia (1997-2013) using a self-exciting spatiotemporal point process model**

**Short title: A self-exciting spatiotemporal point process model to estimate koala density**

Ravi Bandara Dissanayake^1 *^, Emanuele Giorgi^2^, Mark Stevenson^3^, Rachel Allavena^1^, Joerg Henning^1^

^1^ School of Veterinary Science, The University of Queensland, Gatton, Qld, Australia

^2^ Lancaster Medical School, Lancaster University, Lancaster, UK

^3^ Faculty of Veterinary and Agricultural Sciences, University of Melbourne, Parkville, Vic., Australia

*Correspondence: Ravi Bandara Dissanayake, School of Veterinary Science, The University of Queensland, Gatton, Qld 4343, Australia. Email: [r.dissanayake@uq.net.au](mailto:r.dissanayake@uq.net.au)

| 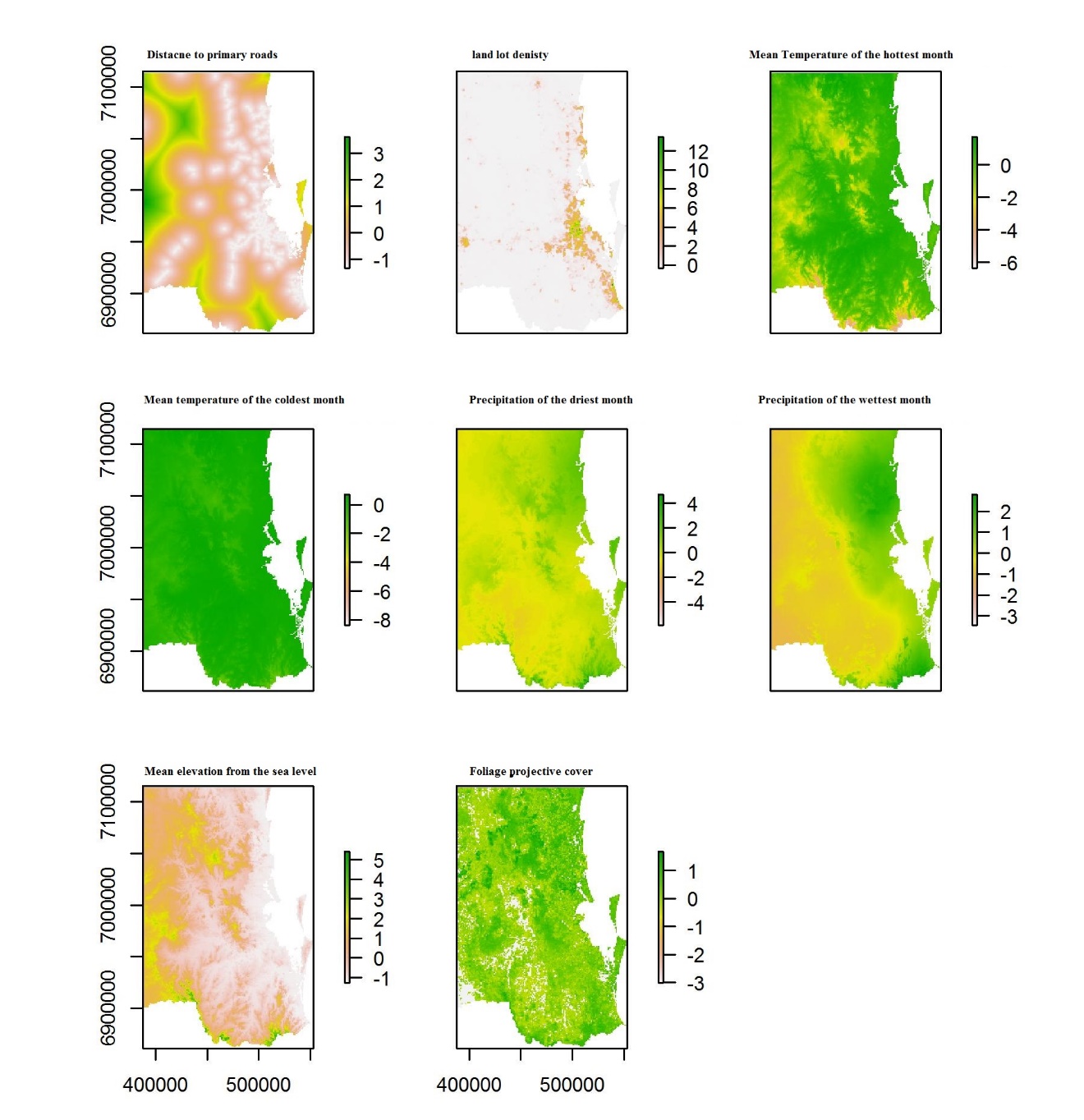 |
| --- |
| **Supplementary Figure 1**: Raster maps of standardised covariates used in spatiotemporal point process model to estimate koala density (koalas per km^2^) in South-East Queensland, 1997-2013. |


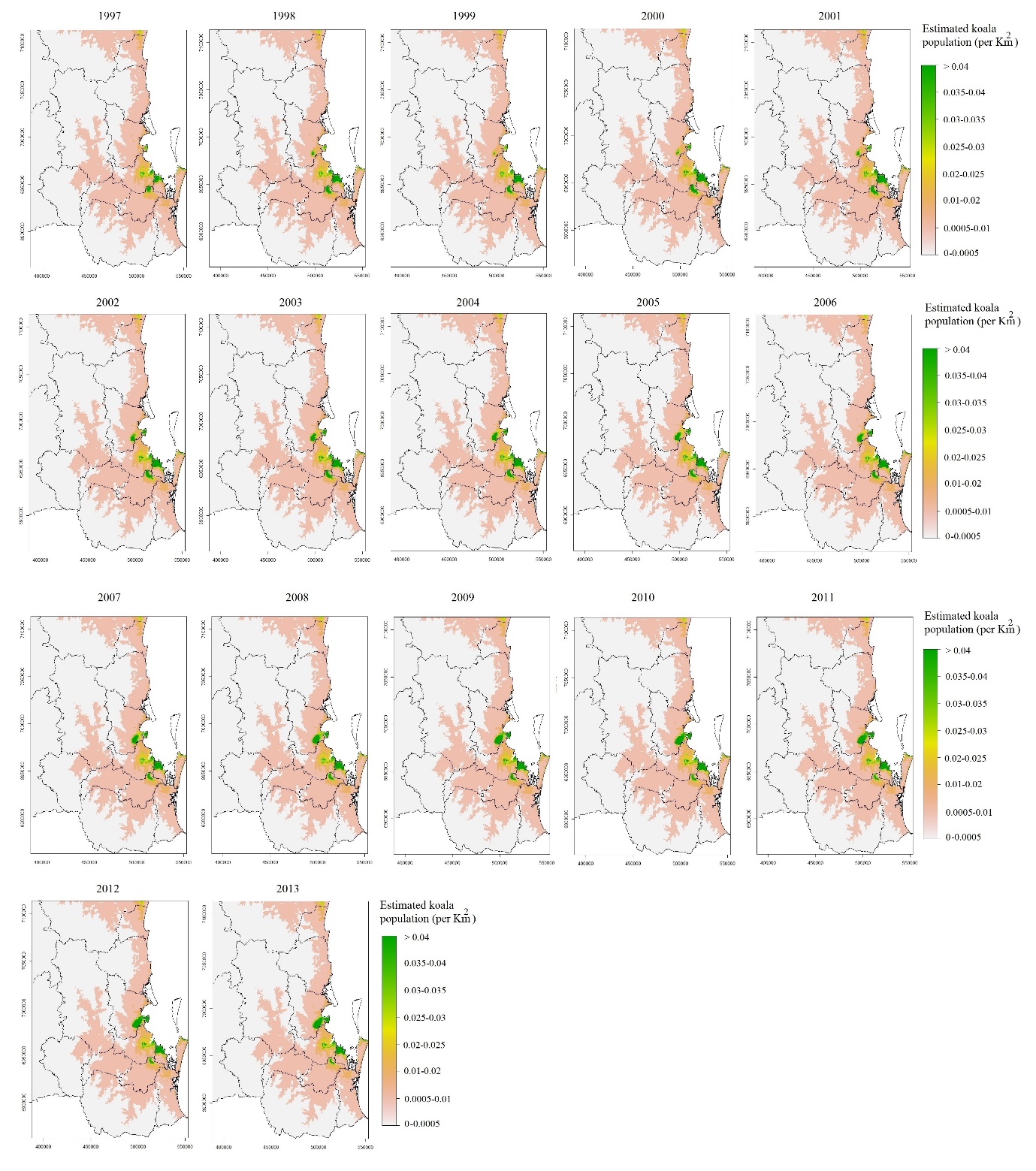


**Supplementary Figure 2:** Raster maps showing estimated koala population densities (koalas per km^2^) across South-East Queensland from 1997-2013. Estimates were derived from a spatiotemporal point process model using koala sightings data ($n$ = 6,580).

| **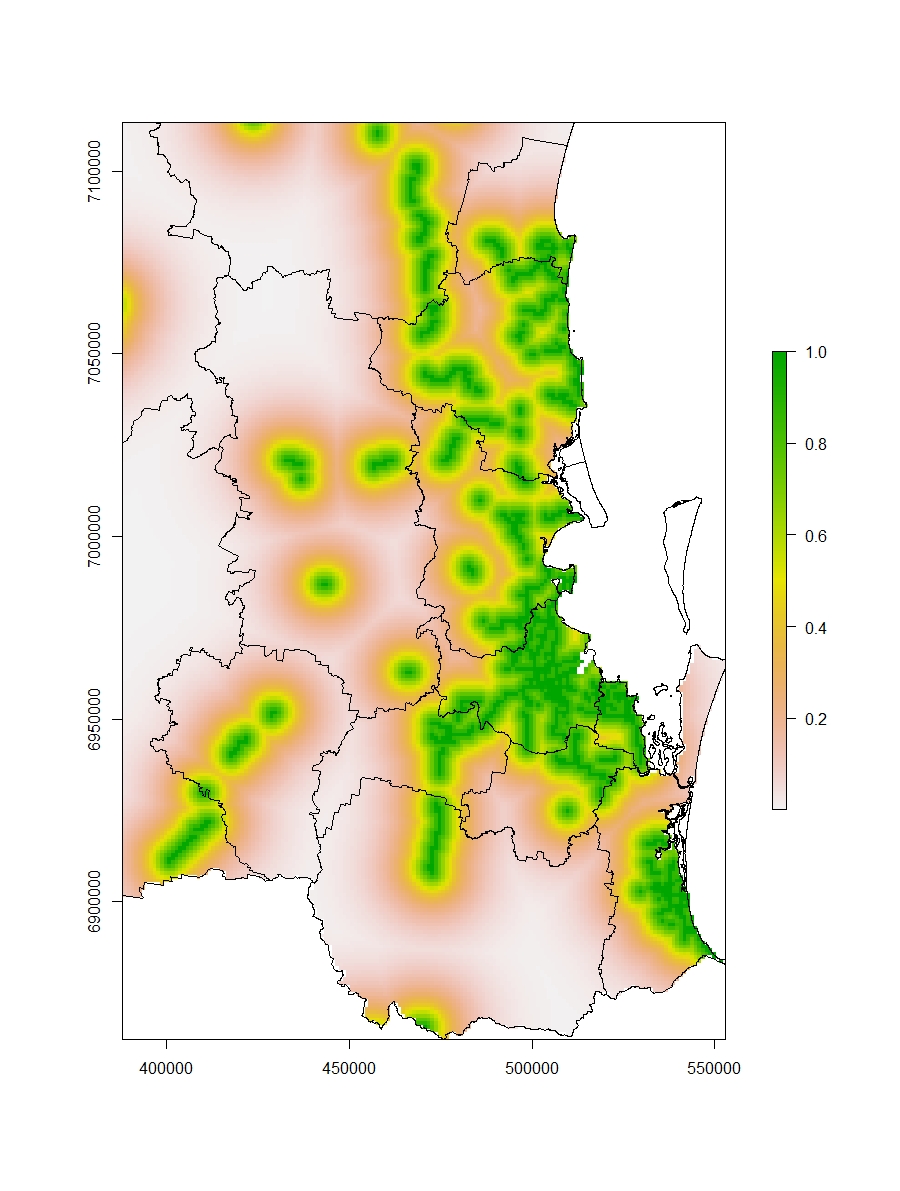** |
| --- |
| **Supplementary Figure 3:** Raster map showing the estimated detection bias for koala sightings reported in South-East Queensland, 1997-2013. |
