# Supplementary material for: Estimating koala density from incidental koala sightings in South‐East Queensland, Australia (1997–2013), using a self‐exciting spatio‐temporal point process model
Source: Ecol Evol. 2021 Sep 17;11(20):13805–14. doi: 10.1002/ece3.8082 (PMC8525080; doi:10.1002/ece3.8082)
Supplement: Supplementary file 2 — Table S1 [file ECE3-11-13805-s002.docx]

**Estimating koala density from incidental koala sightings in South-East Queensland, Australia (1997-2013) using a self-exciting spatiotemporal point process model**

**Short title: A self-exciting spatiotemporal point process model to estimate koala density**

Ravi Bandara Dissanayake^1 *^, Emanuele Giorgi^2^, Mark Stevenson^3^, Rachel Allavena^1^, Joerg Henning^1^

^1^ School of Veterinary Science, The University of Queensland, Gatton, Qld, Australia

^2^ Lancaster Medical School, Lancaster University, Lancaster, UK

^3^ Faculty of Veterinary and Agricultural Sciences, University of Melbourne, Parkville, Vic., Australia

*Correspondence: Ravi Bandara Dissanayake, School of Veterinary Science, The University of Queensland, Gatton, Qld 4343, Australia. Email: [r.dissanayake@uq.net.au](mailto:r.dissanayake@uq.net.au)

**Supplementary Table 1.** Estimated spatial covariate coefficients (95% confidence intervals) from a spatiotemporal point process model used to estimate koala population densities in South-East Queensland between 1997 and 2013.

| **Spatial covariate** | **Estimate coefficient (95% CI)** |
| --- | --- |
| Distance to primary roads (meters) | -0.00015 (-0.00015, -0.00013) |
| Land lot density (number of lots per square kilometre ) | 0.00053 (0.00047, 0.00059) |
| Mean temperature of the hottest month (centigrade) | -0.00516 (-0.00677, -0.00355 ) |
| Mean temperature of the coldest month (centigrade) | 2.56832 (2.43931, 2.69732) |
| Precipitation of the driest month (millimetres) | -0.06207 (-0.07396, -0.05017) |
| Precipitation of the wettest month (millimetres) | -1.01564 (-1.09671, -0.93457) |
| Mean elevation from the sea level (meters) | -0.01119 (-0.01236, -0.01001) |
| Foliage projective cover (proportion) | -0.00165 (-0.00243, -0.00087) |
| **theta_1 (**mating season**)** | 2.00564 (1.92210, 2.09281) |
| **theta_2 (**non mating season**)** | 2.02965 (1.94209, 2.12116) |
| **Phi (**Scale interaction**)** | 1.63313 (1.52694, 1.74669) |
